# The challenges of COVID‐19 Delta variant: Prevention and vaccine development

**DOI:** 10.1002/mco2.95

**Published:** 2021-10-19

**Authors:** Xuemei He, Cai He, Weiqi Hong, Kang Zhang, Xiawei Wei

**Affiliations:** ^1^ Laboratory of Aging Research and Cancer Drug Target State Key Laboratory of Biotherapy and Cancer Center National Clinical Research Center for Geriatrics West China Hospital Sichuan University Chengdu Sichuan P. R. China; ^2^ Center for Biomedicine and Innovations Faculty of Medicine Macau University of Science and Technology Taipa Macau P. R. China

**Keywords:** Delta variant, mutation, SARS‐CoV‐2, spike, vaccine

## Abstract

Several SARS‐CoV‐2 variants have emerged since the pandemic, bringing about a renewed threat to the public. Delta variant (B.1.617.2) was first detected in October 2020 in India and was characterized as variants of concern (VOC) by WHO on May 11, 2021. Delta variant rapidly outcompeted other variants to become the dominant circulating lineages due to its clear competitive advantage. There is emerging evidence of enhanced transmissibility and reduced vaccine effectiveness (VE) against Delta variant. Therefore, it is crucial to understand the features and phenotypic effects of this variant. Herein, we comprehensively described the evaluation and features of Delta variant, summarized the effects of mutations in spike on the infectivity, transmission ability, immune evasion, and provided a perspective on efficient approaches for preventing and overcoming COVID‐19.

## Graphical abstract

SARS‐CoV Delta (B.1.617.2) variant has been classified as variants of concern (VOC) by World Health Organization (WHO). The reproductive number (R0) of SARS‐CoV‐2 wild type and Delta variant is 2.3‐5.7 and 5‐8, respectively. Patients infected by Delta variant exhibit shorter mean generation time and mean serial interval, higher virus load and hospitalization rate compared to those infected by wild‐type SARS‐CoV‐2.

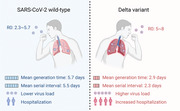

## 1 EMERGENCE OF SARS‐COV‐2 B.1.617 LINEAGES

Coronavirus disease 2019 (COVID‐19) is a highly contagious viral disease caused by severe acute respiratory syndrome coronavirus 2 (SARS‐CoV‐2).^1^ As of September 4, 2021, this pandemic has resulted in 4.5 million deaths worldwide according to the World Health Organization (WHO) (https://www.who.int/). With the joint efforts of the global community, vaccine protection and effective supervision of SARS‐CoV‐2 positive individuals greatly reduced COVID‐19 hospitalizations and deaths. However, in recent months when B.1.617 lineages, especially B.1.617.2 variant (also named Delta) rapidly spread, even though more than five billion vaccine doses have been administered globally (https://www.who.int/), thousands of new cases are diagnosed every day. Therefore, it is necessary to have a more detailed understanding of the SARS‐CoV‐2 Delta variant.

**FIGURE 1:**
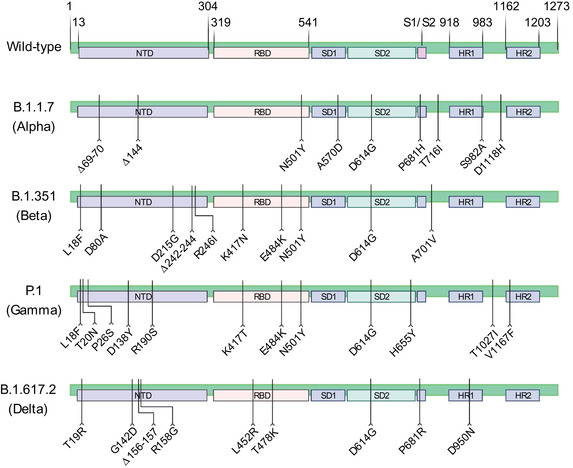
The schematic overview of mutations in spike in Alpha, Beta, Gamma, and Delta variants. The Alpha (B.1.1.7) variant has deletions at site 69, 70, and 144 in spike. And seven substitutions in spike are N501Y, A570D, D614G, P681H, T716I, S982A, and D1118H. The Beta (B.1.351) variant has a deletion at site 242 to 244 and another nine substitutions (L18F, D80A, D215G, R246I, K417N, E484K, N501Y, D614G, and A701V) in spike. The Gamma (P.1) variant harbors 12 mutations in spike, and they are L18F, T20N, P26S, D138Y, R190S, K417T, E484K, N501Y, D614G, H655Y, T1027I, and V1167F. The Delta (B.1.617.2) variant has a deletion at site 156, 157, and 8 substitutions (T19R, G142D, R158G, L452R, T478K, D614G, P681R, and D950N) in spike

As a typical RNA virus, the pace of mutation is about 10^−4^ replacement per site for every year.^2^, ^3^ The genome of SARS‐CoV‐2 is relatively unstable compared to other RNA viruses.^2^ The mutations in the genome lead to modifications in phenotype including different antigens, changes in transmissibility or virulence. Variants with higher transmissibility and infectivity, drug resistance, or immune evasion are more likely to be preserved in the selection. Therefore, several SARS‐CoV‐2 variants have emerged to replace the pre‐existing SARS‐CoV‐2 variants and spread globally. With competitive advantages over their ancestors, the variants tend to be dominant in many cases. Until now, the existing variants have been designated as variants of concern (VOC) and variants of interest (VOI) by WHO. VOC contains Alpha (B.1.1.7), Beta (B.1.351), Gamma (P.1), Delta (B.1.617.2) variants (Figure 1) and their sublineages while VOI contains B.1.525 (Eta), B.1.526 (Iota), B.1.617.1 (Kappa), C.37 (Lambda), and B.1.621 (Mu) variants (https://www.who.int/en/activities/tracking‐SARS‐CoV‐2‐variants). By August 24, 2021, cases infected by Alpha, Beta, Gamma, and Delta variants have been reported in 192, 141, 86, and 163 countries, respectively (https://apps.who.int/iris/bitstream/handle/10665/344560/CoV‐weekly‐itrep24Aug21‐eng.pdf?sequence=1&isAllowed=y).

In early 2021, the variant responsible for the outbreak of COVID‐19 in India was initially called “double mutant” due to E484Q and L452R mutations in spike.^4^ This name “double mutant” was soon modified as B.1.617 since it had a group of sequence clusters with common L452R, D614G, and P681R mutations.^5^, ^6^ Notably, the B.1.617 lineages are not homogeneous. Some multiple mutations, such as T19R, G142D, or D950N, frequently appear together in a lineage and also can be detected in other sublineages at a lower frequency.^7^ With the sequencing of these variants, the first sequence cluster was identified in India carrying T19R, G142D, L454R, E484Q, D614G, P681R, and D950N substitutions in spike.^5^ The emergence of Q1071H mutation produced the B.1.617 lineages variants into three new subclusters, and the first identified sublineage was B.1.617.1 variant (Kappa), followed by B.1.617.2 variant (Delta) and B.1.617.3 variant.^5^, ^6^ B.1.617.1 variant was reclassified to a VOI by WHO on April 4, 2021, because of its increased transmissibility, but the global prevalence seemed to be declined. Delta variant was first detected in October 2020 in India and further determined in March 2021 in the United States.^8^ In India, it spread rapidly and the proportion of Delta variant in all sequenced samples increased from 4.0% on March 8, 2021 to 30.4% on March 29, 2021 according to GISAID (www.gisaid.org). The WHO classified the Delta variant as a VOI on April 4, 2021. Later, because of its astonishing transmissibility and infectivity, the Delta variant displaced other pre‐existing lineages and its percentage in all circulating viruses significantly raised to 89.8% on May 10, 2021 among sequences in India (www.gisaid.org). Delta variant caused 17 million COVID‐19 patients including reinfections during March‐May 2021.^9^ These results prompted the WHO to classify it as a VOC on May 11, 2021. Presently, B.1.617.3 is neither a VOC nor a VOI because of its low prevalence. In short, the B.1.617 and its sublineages are mainly responsible for the second wave of infections in India, resulting in more than 30 million COVID‐19 patients and 4,00,000 deaths in India alone.^10^

## 2 THE FEATURES OF COVID‐19 CAUSED BY DELTA VARIANT

**FIGURE 2:**
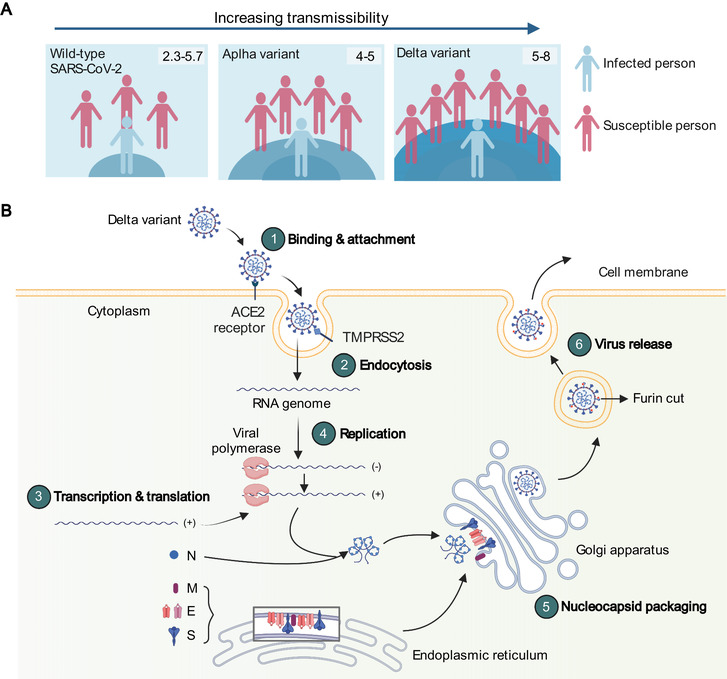
The high transmissibility and life cycle of the Delta variant. (A) The R0 of wild‐type SARS‐CoV‐2 virus, Alpha variant, Delta variant is 2.3‐5.7, 4–5, 5–8, respectively. (B) A simplified diagram to show how Delta variant enters and exits cells. The spike of Delta variant binds to ACE2 on the host cell. The spike was cleaved by transmembrane serine protease 2 (TMPRSS2) to expose the parts that are necessary for the fusion of the virus and host cell membranes. And the virus shoots its RNA genome into the host cell and viral RNA and proteins are synthesized. Then, the newly made molecules are processed and packaged in Golgi apparatus to assemble into a complete virus particle. The spike is cut by furin that is a host enzyme, which prepares the virus to strike another cell. A higher proportion of snipped spike proteins in variant may be involved in higher infectivity. N: nucleocapsid protein, M: membrane protein, E: envelope protein, S: spike protein

The reproductive number (R0) of SARS‐CoV‐2 wild type has been estimated to be as high as 2.3‐5.7 (Figure 2A)^11^, ^12^ Significantly, recent studies have revealed the R0 of Delta variant is up to 5–8, higher than Alpha, Beta, and Gamma variants about 55% (95% CI: 43–68), 60% (95% CI: 48–73), and 34% (95% CI: 26–43), respectively,^9^, ^13^, ^14^, ^15^ indicating its prominent transmissibility. The Delta variant has replaced the pre‐existing lineages and has become the most dominant variant in India and further spreads to 163 countries within a few months. When India was struggling against COVID‐19 cases, a traveller returned from India resulted in 77% of sequenced circulating viruses identified as Delta variant between June 2 and 9 in the United Kingdom where Alpha variant was initially prevalent.^7^ Meanwhile, the transmission advantage of the Delta variant estimated in France is 79% higher than Alpha variant.^16^ As of the early of August 2021, the proportion of Delta variant in circulating virus sequences also accounts for more than 90% over the world (www.gisaid.org), which triggers a new wave of global infection.

The life cycle of the Delta variant in host cells is shown in Figure 2B. The mutant spike of the Delta variant mediates more efficient cell entry and enhanced syncytium formation, which probably implicates high virus load and severe disease.^17^ Mlcochova et al.^17^ reported that Delta variant had a higher replication efficiency in both 3D airway organoid and human airway epithelial organoids compared to Alpha variant. In COVID‐19 patients, the time interval from exposure to the first PCR‐positive test has shortened from 6 days during the 2020 epidemic to 4 days in the recent Delta variant epidemic, indicating the shorter incubation period of the Delta variant.^18^ Another outbreak in Guangdong Province in China also exerts a similar incubation period.^19^ The mean generation time and mean serial interval are 2.9 and 2.3 days, respectively, both of which are shorter than that of SARS‐CoV‐2 wild type (2.9 vs 5.7 for mean generation time and 2.3 vs 5.5 for mean serial interval).^19^, ^20^ Additionally, 64.7% (44/68) of transmission events by Delta variant occurred during the presymptomatic phase. But this percentage was 59.2% in the first outbreak in Hubei Province.^20^ More importantly, the viral loads of Delta variant infections were on average approximately 1000 times greater than infections in the initial epidemic wave.^18^ In Scotland, the Delta variant was found mainly in younger people, and the risk of hospitalization was approximately doubled in those infected with Delta variant compared to Alpha variant infection.^21^ Likewise, a significantly increased risk of emergency care attendance or hospitalization was observed in the United Kingdom.^16^ Taken together, these transmission parameters suggest the probability of Delta variant becoming the dominant circulating lineages and indicate a significant challenge to battle against Delta variant.

Several properties of the Delta variant have been analyzed to explain why Delta broke through a variety of variants. On the one hand, the Delta variant spike fuses more efficiently with target cells with low human angiotensin‐converting enzyme 2 (hACE2) level than other variants, and its pseudovirus infects target cells significantly faster than other variants.^22^ This evolutionary optimization of efficient fusion may explain why Delta variant can rapidly attack more cells and spread from person to person in a rather short exposure time. On the other hand, structural analysis of receptor‐binding domain RBD‐ACE2 interface reveals that a total of 17 residues in RBD interact with 20 residues of ACE2 to formulate networks of hydrophilic interactions that involve in the virus‐receptor engagement.^23^ The Delta spike shares four substitutions (L452R, D614G, P681R, and D950N) with B.1.617 and its sublineage and harbors additional five mutations (T19R, G142D, Δ156‐Δ157, R158G, and T478K).^24^, ^25^ Presently, all the circulating SARS‐CoV‐2 variants evolve based on D614G that is involved in high transmissibility and infectivity but not in disease severity.^26^, ^27^, ^28^, ^29^, ^30^, ^31^, ^32^ L452 residue is located on the edge of the receptor‐binding motif (RBM) of RBD and can directly contact with ACE2. It is necessary for the hydrophobicity with L492 that forms another hydrophobic interaction with F490.^5^, ^33^ L452R substitution enhances viral infectivity and fusion efficacy as well as viral replication.^34^ P681 is located at the unique “PRRAR” furin cleavage sites at the S1‐S2 boundary of the spike. It plays an important role in facilitating the transmission of variants between humans.^23^ P681R substitution in spike optimizes the furin cleavage site and enables more efficient spike cleavage by furin, resulting in augmented syncytium formation and consequently enhanced transmissibility and pathogenicity.^35^, ^36^ D950 located in heptad repeat 1 (HR1) of S2 subunit where is a critical site that can influence the refolding of S2. D950 is required for the fusion of SARS‐CoV‐2 and host cell membranes.^22^ D950N mutation eliminates a negative charge, which shows no obvious influence on spike structure but might enhance fusogenicity of the Delta spike.^22^ Collectively, these mutations in the Delta spike work together and finally improve its fitness advantages, pathogenicity, and infectivity.

## 3 IMMUNE EVASION OF DELTA VARIANT

The double mutations of E484Q and L452R in the spike of B.1.617 lineages had caused global concerns since E484 mutation appeared in the rapidly spreading variants Beta and Gamma and was associated with immune evasion.^5^, ^37^ Previous studies reported that E484 residue was theoretically recognized as a repulsive residue at the RBD‐ACE2 interface, and mutation at this site would be beneficial to RBD‐ACE2 binding.^38^ More importantly, E484 and L452 mutations are involved in impaired neutralization by antibodies.^39^, ^40^ Therefore, B.1.617, B.1.617.1, and B.1.617.3 variants with E484 and L452 mutations were initially considered to be strongly resistant to the immune response of the body. However, recent evidence has revealed that the majority of sera from convalescents and vaccinated individuals can neutralize B.1.617 and B.1.617.1 variants, and the reduction of neutralizing ability is limited to 2‐fold. Delta variant is 5.7‐ and 8‐fold less sensitive to the sera from convalescent and vaccinated individuals compared to the SARS‐CoV‐2 wild type.^17^, ^41^, ^42^, ^43^ The sera from COVID‐19 convalescents were 4‐fold less potent against Delta variant when compared with that of the Alpha variant, which was similar to the Beta variants.^7^ Moreover, sera from individuals vaccinated one dose of Pfizer or AstraZeneca vaccine showed poor neutralization against the Delta variant. A total of 95% of individuals who obtained full vaccination generated a neutralizing response against the Delta variant but the titers were 3‐ to 5‐fold lower than against the Alpha variant.^7^ In several clinical trials, the adjusted vaccine effectiveness (VE) against infection among those fully vaccinated declined about 20% for mRNA vaccine and adenovirus vector vaccine ChAdOx1.^21^, ^44^ The effectiveness of the present COVID‐19 vaccines against the Delta variant is summarized in Table 1. Notably, a recent study demonstrated that there was no difference in viral loads between unvaccinated individuals and fully vaccinated individuals, who also can be infected with Delta variant, 33% (26/79) of who infected with Delta variant showed *Ct* value below 20.^45^

**TABLE 1:** The effectiveness of current COVID‐19 vaccines against the Delta variant

Name (company)	Antigen	VE against wild type	Measure outcome	1st Dose VE (95% CI)	2^st^ Dose VE (95% CI)	Country	References (Date)
BNT162b2 (Pfizer‐BioNTech)	Full‐length spike protein with proline substitutions	95%	Symptomatic COVID‐19	35.6% (22.7‐46.4)	88% (85.3‐90.1)	UK	^44^ (July 21, 2021)
			Symptomatic infection	56% (45‐64)	87% (64‐95)	Canada	^67^ (July 3, 2021)
			Hospitalization or death	78% (65‐86)	—		
			Hospitalization	94% (46‐99)	96% (86‐99)	UK	^68^ (June 14, 2021)
			Documented infection	30% (17‐41)	79% (75‐82)	Scotland	^21^ (June 14, 2021)
			Symptomatic infection	33.2% (8.3‐51.4)	87.9% (78.2‐93.2)	UK	^58^ (May 20, 2021)
mRNA‐1273 (Moderna)	Full‐length spike protein with proline substitutions	94.1%	Symptomatic infection	72% (57‐82)	—	Canada	^67^ (July 3, 2021)
			Hospitalization or death	96% (72‐99)	—		
ChAdOx1 (AZD1222) (AstraZeneca)	Replication‐deficient chimpanzee adenoviral vector with the SARS‐CoV‐2 spike protein	62.1% (two standard doses) 90% (a low dose followed by a standard dose)^69^	Symptomatic COVID‐19	30% (24.3‐35.3)	67% (61.3‐71.8)	UK	^44^ (July 21, 2021)
			Symptomatic infection	67% (44‐80)	—	Canada	^67^ (July 3, 2021)
			Hospitalization or death	88% (60‐96)	—		
			Hospitalization	71% (51‐83)	92% (75‐97)	UK	^68^ (June 4, 2021)
			Documented infection	18% (9‐25)	60% (53‐66)	Scotland	^21^ (June 14, 2021)
			Symptomatic infection	32.9% (19.3‐44.3)	59.8% (28.9‐77.3)	UK	^58^ (May 20, 2021)

Data were collected from WHO and PubMed.

VE, vaccine effectiveness; UK, the United Kingdom.

Meanwhile, the Delta variant exerts resistance to some anti‐N‐terminal domain and anti‐RBD therapeutic monoclonal antibodies for COVID‐19 including Bamlanivimab.^46^ But three other clinically approved monoclonal antibodies Etesivimab, Basirivimab, and Imdevimab preserved neutralization ability against the Delta variant. Besides, using a cocktail of multiple neutralizing antibodies that recognize and bind to different and nonoverlapping epitopes of the spike could restrict the potential loss of single antibody‐mediated neutralization and minimize the possible generation of viral escape mutants.^47^, ^48^

The RBM epitopes in RBD that overlap with ACE2 are immunodominant. T478, E484, and L452 residues are located in immunodominant RBM epitopes and could influence the binding ability by antibodies from serum or some therapeutic antibodies.^35^, ^49^ L452R substitution abolishes the hydrophobic interaction with I and V105 residues of the antibody's heavy chain, resulting in impaired neutralization of RBD‐specific mAbs.^5^, ^50^ Besides, L452R mutation makes a different conformation of epitope peptide, which weakens binding affinity of spike and ACE2 and reduces suboptimal recognition by T‐cell receptor and thereby escape human leukocyte antigen‐restricted cellular immunity.^34^, ^51^ The T478K is a unique substitution in the Delta variant. It falls within the epitope region of potential “Class 1″ neutralizing monoclonal antibodies that bind the spike protein in the open conformation.^52^ However, the mechanism of T478K mutation affecting immune escape remains unclear. Further studies are required to determine its impact. Nonetheless, these novel mutations, including L452R, T478K, and P681R should be seriously considered when developing next‐generation vaccines or monoclonal antibodies in the future.

## 4 PERSPECTIVES OF STRATEGIES AGAINST DELTA VARIANT

### 4.1 Physical protection

WHO has advocated some precautions to protect us from COVID‐19, such as cleaning hands, wearing a mask, keeping rooms well ventilated, etc., especially for healthcare workers (HCWs) who are not only in close contact with COVID‑19 patients, but also in a poorly ventilated environment. Previous studies have shown that the infection rates of SARS‐CoV‐2 among HCWs range 2.2–44% which is significantly higher than that of the general population.^53^, ^54^ Therefore, FFP2/3 respirators or similar respirators are recommended to replace surgical masks when HCWs contact with confirmed or suspected COVID‑19 patients to avoid the secondary transmission, especially in the face of highly infectious variants as Delta.^54^, ^55^, ^56^ Moreover, workers with occupational exposure to SARS‐CoV‐2 should be encouraged to conduct frequent tests to limit virus spread.

### 4.2 Optimizing vaccination program

Studies have demonstrated that pre‐existing cellular immunity in people infected by other coronaviruses might contribute to the different disease severity between COVID‐19 patients,^57^ which might partly explain the fully vaccinated individuals exert more mild‐moderate phenotypes of COVID‐19 compared to unvaccinated individuals. Similarly, although reduction of VE against Delta variant has been observed in several studies, the current vaccines remain protective against Delta variant.^58^ For example, household members who received at least one dose of vaccine were 50% less likely to be infected by SARS‐CoV‐2 than unvaccinated members.^59^ Among outbreak‐associated COVID‐19 patients in central Oklahoma, 85% (40/47) had never received any COVID‐19 vaccine doses, and 6% (3/47) and 9% (4/47) had received one dose and two doses of Moderna or Pfizer‐BioNTech vaccines.^15^ These results indicate that vaccination remains an effective measure to avoid Delta variant infection. The development of specific vaccines based on the Delta spike can effectively improve the protection efficiency of vaccines. Before the use of Delta‐specific vaccine, optimization of our vaccination program may be an effective strategy to enhance the VE against Delta variant. Using a combination of a DNA vaccine and a recombinant S1 subunit vaccine induced high levels of nAbs as well as strong T‐cell immune responses to protect rhesus macaques after the challenge of SARS‐CoV‐2 viruses, which is better than administration DNA or protein vaccine alone.^60^ And Georg Behrens, an immunologist at Hanover Medical School in Germany, pointed out that vaccination with different types of vaccines could trigger a better overall response to increase the effectiveness.^61^

Moreover, oral and nasal mucosae serve as the primary defense to prevent pathogens from entry and infection.^62^ Evidence and lessons from SARS‐CoV and MERS‐CoV indicate that activation mucosal immune response is a comprehensive strategy to suppress SARS‐CoV‐2 infection, especially in the face of Delta variant that shows less resistance to vaccines compared to Beta variant but exerts prominent transmissibility and infectivity.^63^, ^64^, ^65^ Therefore, developing COVID‐19 vaccines that could elicit a strong immune response at both mucosal sites and systemic circulation might be a promising approach to suppress the infection and transmission of the Delta variant.

## 5 CONCLUSIONS

Mutations will occur in the future due to natural selection, random genetic drift, or influence of the environment.^66^ Some other variants have appeared, such as Lambda (C.37), Mu (B.1.621), Theta (P.3), etc. Effective caution and protective measures should be taken to ensure safe contacts to suppress the spread of SARS‐CoV‐2. Genomic surveillance of COVID‐19 patients, especially vaccine breakthrough cases, will be essential to monitor the evolution of SARS‐CoV‐2. Developing safe and effective vaccines and a global vaccination programme are necessary strategies to tackle or eliminate the COVID‐19 pandemic.

## CONFLICT OF INTEREST

The authors declare no conflict of interest.

## AUTHORS’ CONTRIBUTIONS

Xiawei Wei and Kang Zhang conceived the study and revised the manuscript. Xuemei He, Cai He, and Weiqi Hong wrote the article.

## ETHICS STATEMENT

Not applicable.

## Data Availability

The data included in this study are available upon request from the corresponding author.
